# A Simplified Technique for All-Inside Tibial Socket Retrograde Drill Guiding Using a 2- to 3.5-mm Concentric Cannula Without the All-Inside Tibial Guide Ring

**DOI:** 10.1016/j.eats.2024.103177

**Published:** 2024-08-23

**Authors:** Yizhong Peng, Wenbo Yang, Wei Yu, Chunqing Meng, Hong Wang, Wei Huang

**Affiliations:** Department of Orthopaedics, Union Hospital, Tongji Medical College, Huazhong University of Science and Technology, Wuhan, China

## Abstract

The all-inside anterior cruciate ligament (ACL) technique is a minimally invasive surgical procedure that has gained popularity due to its reduced invasiveness and improved patient outcomes. The establishment of tibial sockets remains a crucial step in ACL reconstruction, which has always been difficult in ACL reconstruction research. For doctors who are not very experienced, this technique of positioning and making sockets requires a special guide ring, and the tunnel entrance is prone to be anterior. Herein, we report a simplified technique using a self-made 2- to 3.5-mm concentric cannula to help surgeons easily master the all-inside technique. Our technique for tibial socket construction does not require the specific tibial guiding ring but uses a traditional tibial guiding ring for full-length tibial tunnel construction. With a 2-mm Kirschner wire and the traditional tibial guiding ring initially locating the tunnel position, the self-made concentric cannula helps combine the Kirschner wire with the guide pin sleeve, thereby impacting the guide pin sleeve into the tibial cortex at a controlled depth. Then, retrograde drilling is performed to create the socket. This technique provides feasible approaches for surgeons to transition from traditional full-long tunnel creation to the semi-long socket construction for the all-inside ACL technique.

The all-inside anterior cruciate ligament (ACL) technique is a less invasive alternative to traditional ACL reconstruction surgery.^1^ The minimally invasive approach reduces postoperative pain, swelling, and complications, leading to faster recovery and improved patient satisfaction.^2^ Over the years, several advancements have been made in the field of minimally invasive ACL reconstruction, focusing on improving the precision, safety, and efficacy.^3^^,^^4^

Numerous studies have reported favorable clinical outcomes following the all-inside ACL technique, with high success rates and low complication rates. Furthermore, long-term follow-up studies have shown that patients who underwent minimally invasive ACL reconstruction had similar functional outcomes and knee stability as those who underwent open surgery.^4^, ^5^, ^6^

The construction of the tibial socket is a critical aspect of this technique, as it directly affects the stability and longevity of the graft-link construct.^7^ One of the key advancements in minimally invasive ACL reconstruction is the development of specialized instruments designed specifically for the all-inside ACL technique, including retrograde drilling pins, guide pin sleeve, and so on.^8^ Thanks to this equipment, the tibial socket can be created to be deep enough to securely hold the graft but not so deep that it compromises the integrity of the tibial plateau, which relies on the guide pin sleeve to control the depth.^9^

Research about ACL reconstruction has mainly focused on the selection of graft and its fixation.^10^ Few have reported the improvement of the surgical process, especially socket construction. Considering the importance of accurate tibial socket construction, we report a simple technique using a 2- to 3.5-mm concentric cannula for retrograde drill guiding without the all-inside tibial guide ring to create a tibial socket.

## Surgical Technique

**Fig 1:**
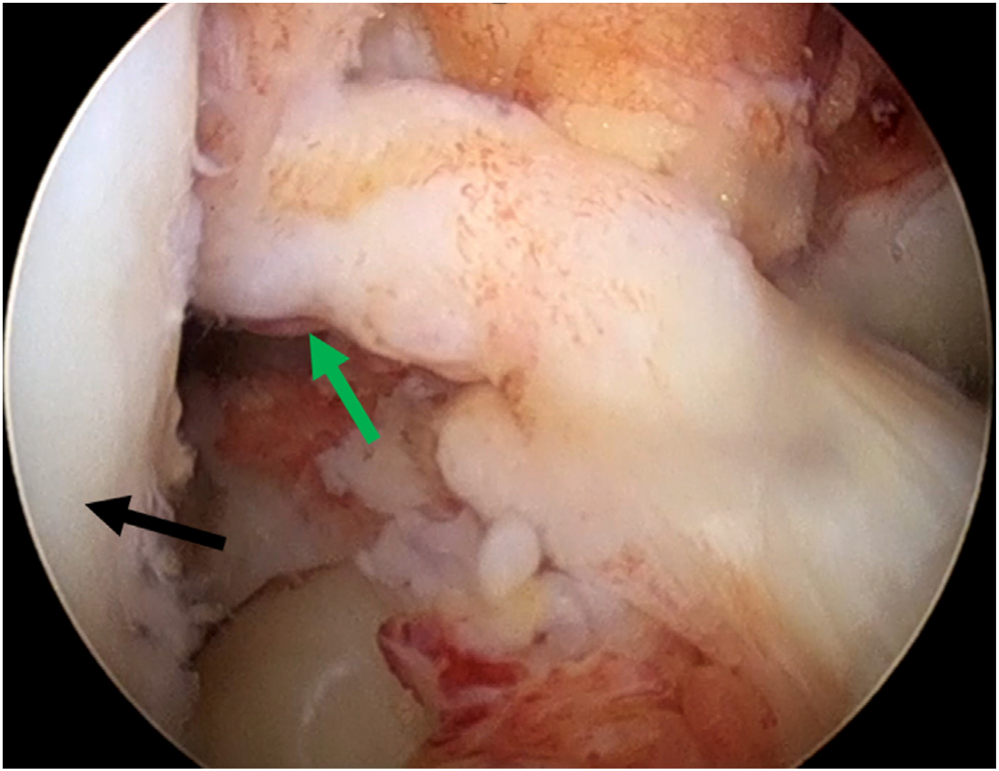
Arthroscopic view from the anterolateral portal to identify the anterior cruciate ligament rupture of the right knee. Green arrows: ruptured anterior cruciate ligament stump. Black: lateral femoral condyle. Patient in supine position with the knee in 90° flexion.

Our surgical technique is described in detail in Video 1. After general anesthesia, the patient is draped in the supine position, and the affected limb is disinfected in a standardized fashion for arthroscopic ACL surgery. The acute proximal ACL tear of the right knee is detected under an anterolateral observation portal (Fig 1).

**Fig 2:**
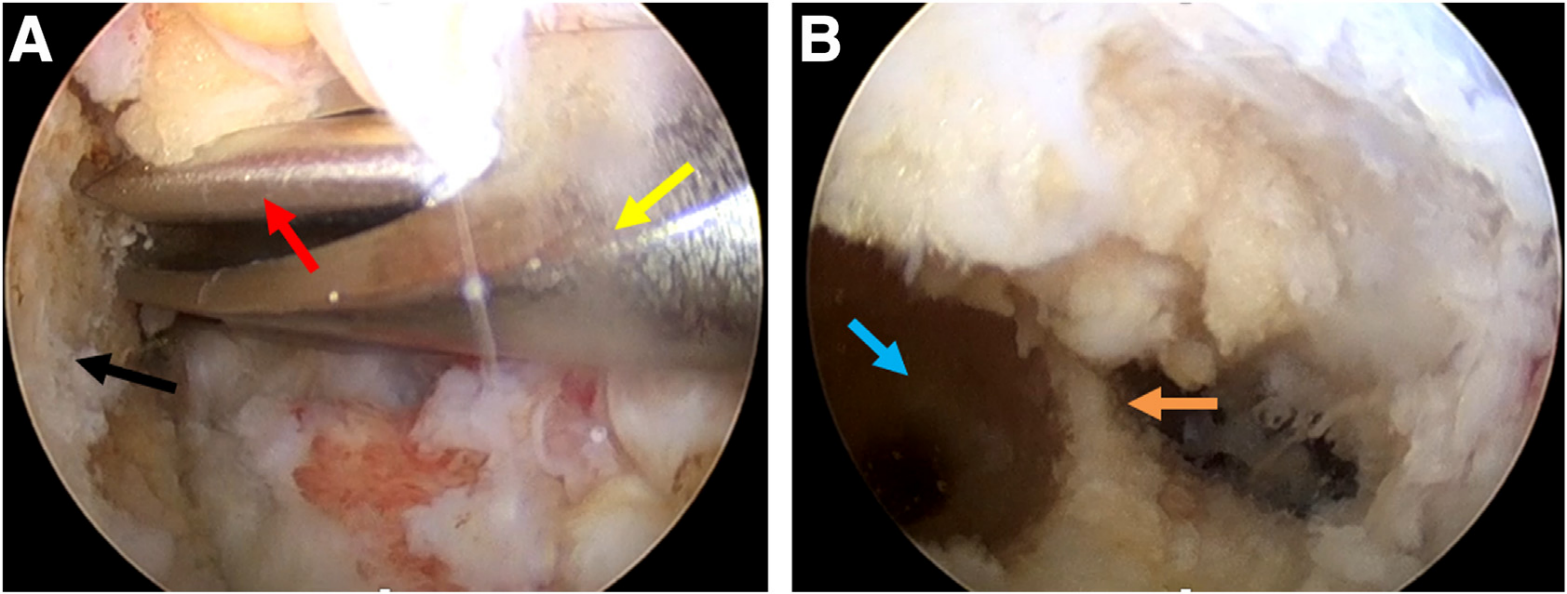
Preparation of the femoral socket with the anterolateral portal for observation and the anteromedial portal for operation. (A) A femoral tunnel is prepared using a femoral offset positioner. (B) The prepared femoral socket and posterior wall are exposed. Black arrow: medial aspect of the lateral femoral condyle. Red arrow: 2-mm Kirschner wire. Yellow arrow: femoral offset positioner. Blue arrow: prepared femoral socket. Orange arrows: posterior wall. The patient is supine with the right knee in 120° flexion.

The semitendinosus tendon is harvested for graft preparation. Under the monitoring of arthroscopy, an offset positioner is placed in from the anteromedial portal (Fig 2A) and a 20-mm-deep bone socket is drilled in the femur with 120° of knee flexion (Fig. 2B).

**Fig 3:**
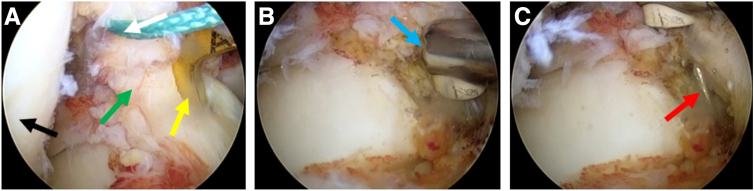
The traditional anterior cruciate ligament (ACL) tibial guide ring guiding the placement of the 2-mm Kirschner wire with the anterolateral portal for observation and the anteromedial portal for operation. (A) The traditional ACL tibial guide ring located in the tibial tunnel. (B) Disposable plasma scalpel removed the soft tissue around the outlet that the Kirschner wire drilled through. (C) Observation of a well-located Kirschner wire. Black: lateral femoral condyle. Green: ruptured ACL stump. Yellow: traditional ACL tibial guide ring. Blue: disposable plasma scalpel. Red: Kirschner wire. The patient is supine with the right knee in 90° flexion.

For tibial socket preparation, the traditional ACL tibial guide ring (Smith & Nephew) is placed on the footprint (Fig 3A) and a 2-mm Kirschner wire is drilled in, with 90° of knee flexion (Fig 3B). Then the tibial guide ring is removed, with the Kirschner wire left (Fig 3C).

**Fig 4:**
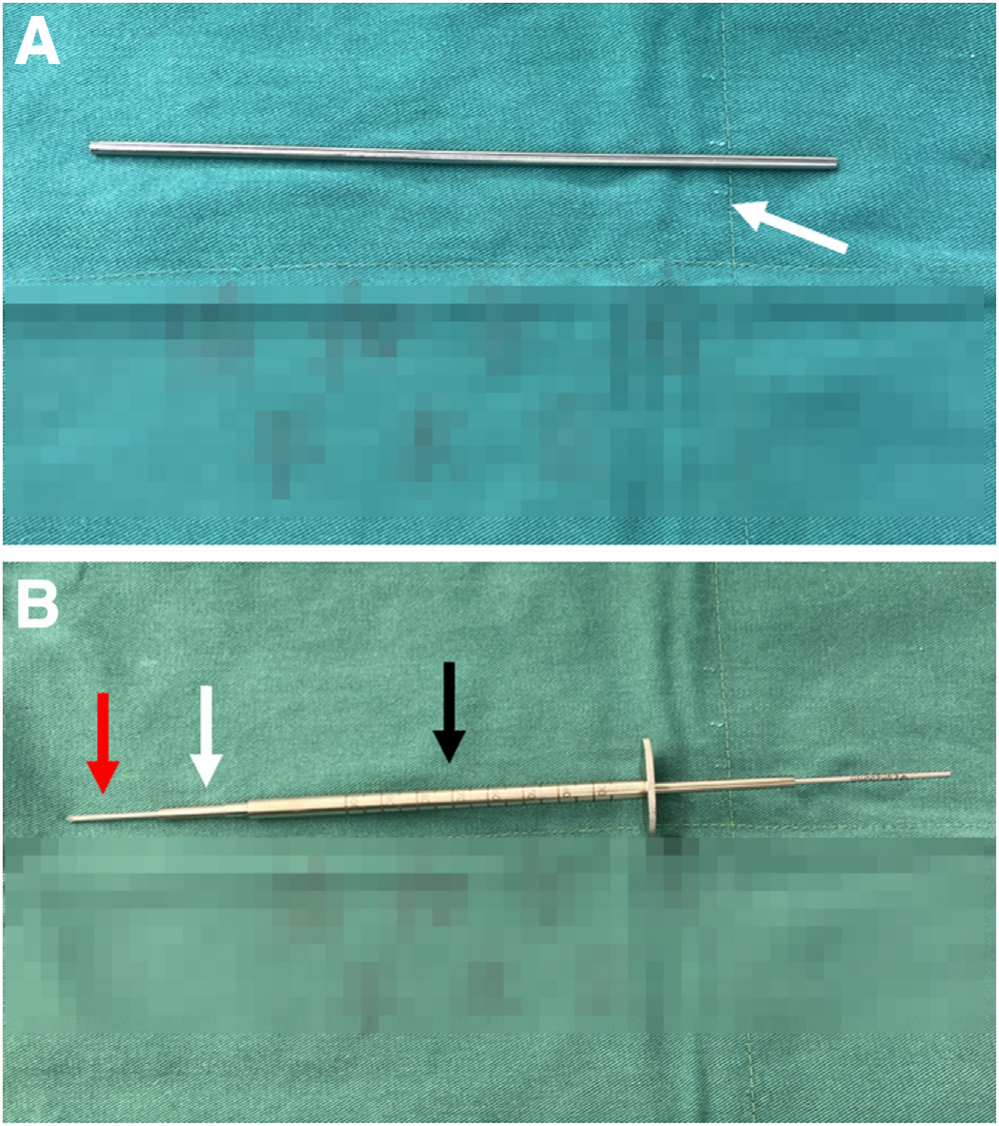
Intuitive diagram of concentric cannula combination. (A) A self-made 2- to 3.5-mm concentric cannula (white arrow). (B) A 2-mm Kirschner wire (red arrow), concentric cannula (white arrow), and 3.5-mm guide pin sleeve (black arrow) nested combination.

**Fig 5:**
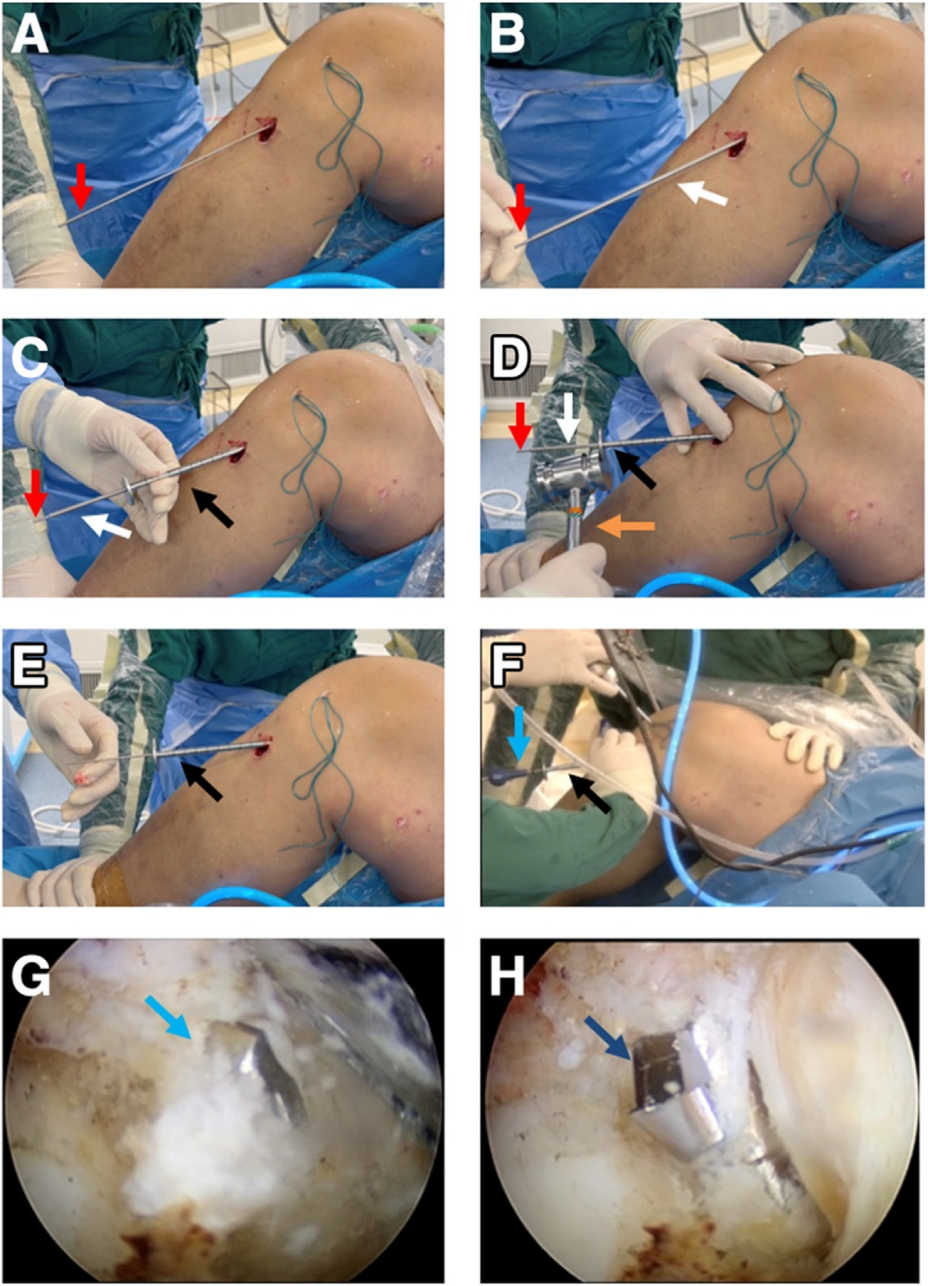
Preparation of a tibial socket using a self-made concentric cannula (no need for all-inside anterior cruciate ligament tibial guide ring). (A) The traditional anterior cruciate ligament tibial guide ring was removed with the 2-mm Kirschner wire left in the bony tract. (B) The self-made 2- to 3.5-mm concentric cannula was put on the Kirschner wire. (C) The guide pin sleeve was put on the self-made concentric cannula sequentially. (D) The cannulated guide pin sleeve with a 7-mm step-off tip was impacted into the bony cortex. (E) The 2-mm Kirschner wire and concentric cannula were removed carefully without intervening with the guide pin sleeve. (F) The 3.5-mm retrograde drill was impacted through the guide pin sleeve. Arthroscopic view from the anterolateral portal to observe the forward drilling into the knee (G) and retrograde drill to create the tibial socket (8 mm in diameter × 30 mm in length) (H). Red arrow: 2-mm Kirschner wire. White arrow: the self-made concentric cannula. Black arrow: the guide pin sleeve with a 7-mm step-off tip. Orange arrow: Hammer; Blue arrow: retrograde drill with forward drilling pattern; Dark blue: retrograde drill with reverse drilling pattern. The patient is supine with the right knee in 90° flexion.

We made a 2- to 3.5-mm concentric cannula (Fig 4A) that can perfectly combine the Kirschner wire and the guide pin sleeve (Fig 4B, Table 1). The 2- to 3.5-mm concentric cannula is inserted along the 2-mm Kirschner wire (Fig 5 A and B), and then a cannulated guide pin sleeve with a 7-mm step-off tip is inserted along the concentric cannula (Fig 5C) and is impacted over the pin into the bony cortex (Fig 5D, Table 1). After confirming that the guide pin sleeve is firmly held in place, the Kirschner wire and the concentric cannula are removed (Fig 5 E and F, Table 1). Next, the 3.5-mm retrograde drill (FlipCutter Drill; Arthrex) is being forward drilled though the guide pin sleeve and reaches to the target footprint (Fig 5G). Then the tibial socket is drilled in a retrograde manner until the drill blade stops advancing when it contacts the step-off tip of the guide pin sleeve (Fig 5H). Subsequently, the retrograde drill is carefully pushed back into the knee, flipped back into guide pin mode, and removed. Then, we complete the preparation of the tibial socket.

**Table 1:** Pearls and Pitfalls of the Simplified All-Inside Tibial Socket Construction

Pearls	Pitfalls
1. With the aid of a self-made concentric cannula, the selection of either a traditional or an all-inside tibial guide ring is more adjustable, according to the usage habits and proficiency of the surgeons.	1. The guide pin sleeve must be hammered into the cortical bone and kept stable; otherwise, it will affect the accuracy of the retrograde drilling into the tibia.
2. A homemade 2- to 3.5-mm concentric cannula combines the Kirschner wire and the guide pin sleeve to ensure the accurate placement of the guide pin sleeve into the tibial bone cortex.	
3. Be careful in the process of pulling out the Kirschner wire and sleeve, so as not to affect the stability of the guide pin sleeve in the tibial bone cortex.

**Fig 6:**
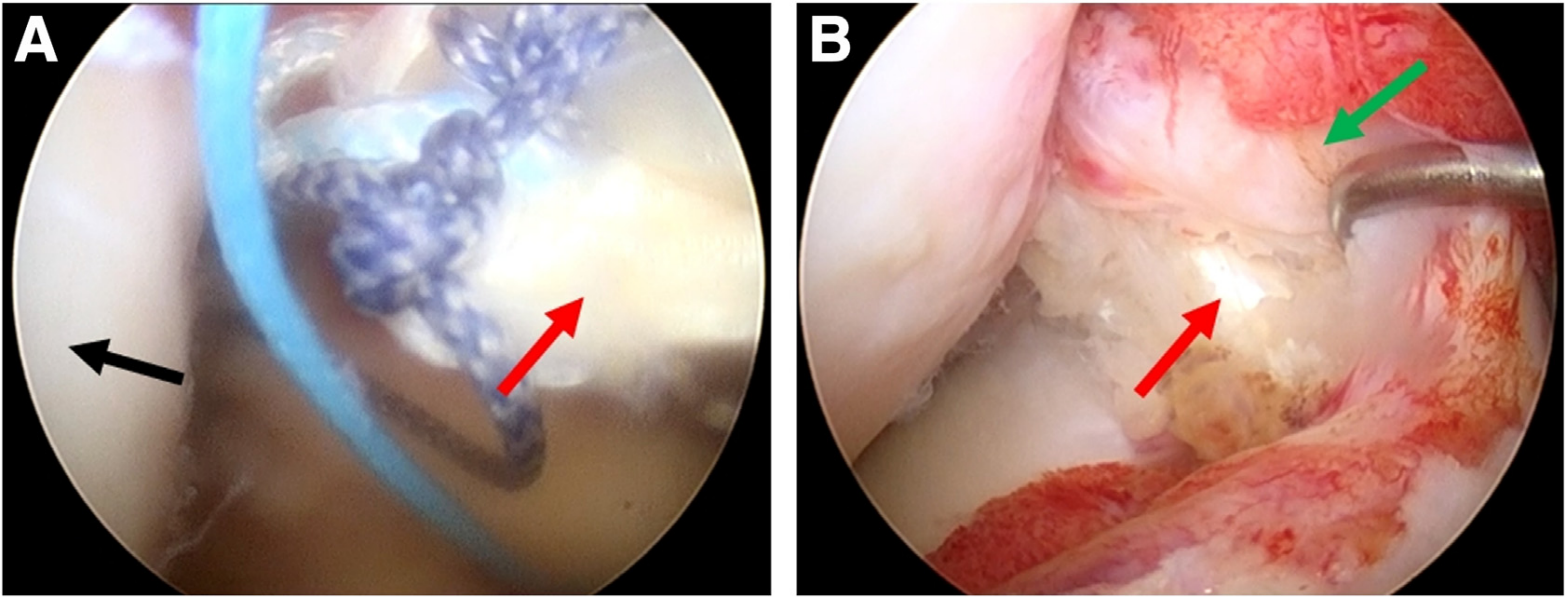
Implantation of the tendon graft into the prepared sockets with the anterolateral portal for observation and the anteromedial portal for operation. (A) The prepared 8-mm × 65-mm annular tendon graft was pulled into the joint. (B) The tendon graft was fixed. Black: lateral femoral condyle. Red: the tendon graft. Green: the ruptured anterior cruciate ligament stump. The patient is supine with the right knee in 90° flexion.

The prepared tendon graft is pulled into the femoral socket first through the anteromedial portal and tensioned once the button flips (Fig 6A). The pull-flip-fill technique is repeated on the tibial side. Then we finish the all-inside ACL reconstruction (Fig 6B).

## Discussion

Although the surgical methods for ACL reconstruction have been constantly evolving, the establishment of femoral and tibial tunnels and the fixation of grafts remain crucial steps in ACL reconstruction.^11^^,^^12^ These aspects have always been hotspots and difficulties in ACL reconstruction research. Compared with complete tibial tunnel techniques, the retro-drilled all-inside tibial tunnel has the advantage of using a single tendon graft, resulting in less early postoperative pain with similar clinical and functional outcomes.^13^ Therefore, many efforts have been made in the field of constructing the tibial socket. In 2002, Morgan et al.^14^ first proposed making a semi-tibial bone tunnel (socket) from inside to outside through a high anteromedial approach. This method does not require a full-layer bone tunnel from the lateral cortex of the bone to the inside of the joint, as in traditional techniques, and both the drilling of the bone tunnel and the fixation of the graft are performed within the joint cavity, so it is named the all-inside technique. Although most doctors believe that the operation is complex and few people dare to try it, this is a milestone for ACL all-inside reconstruction. In 2006, Lubowitz^15^ designed a special tibial retrograde guide for making a tibial and femoral socket. The positioning end inside the joint is equipped with a detachable double reverse drill, which can be used to drill the femoral socket in a forward direction and the tibial socket in an opposite direction. Although the operation still was not simple enough, the making of this tibial socket was a revolutionary change for ACL all-inside reconstruction technology. In 2011, Cerulli et al.^16^ proposed the original all-inside technology and described a retrograde drill with a flippable wing. When the wing is flipped inside the knee joint cavity, it can be used to drill the femoral and tibial socket from inside to outside. Without retrograde drilling, it is almost impossible to make a tibial socket. The retrograde drill with a flippable wing is much simpler than the one designed by Lubowitz^15^ and has played a decisive role in ACL all-inside reconstruction, which has been gradually accepted by many doctors. In 2011, Lubowitz et al.^17^ proposed a second-generation ACL all-inside reconstruction technique. The tibial landmark is located behind the anterior inner bundle endpoint of the ACL, and a semi-long tibial tunnel is made with a flippable wing retrograde drill. Lubowitz et al.^17^ introduced the positioning of tunnels from outside to inside and then used a flippable wing retrograde drill to make a semi-long bone tunnel. The ACL reconstruction surgery we usually carry out is not positioned in this way, so for doctors who are not very experienced, this method of positioning and making tunnels requires a special guide ring, and the tunnel entrance is prone to be anterior. Nevertheless, there is no better way to drill the semi-long tibial tunnel except using a flippable wing retrograde drill. Therefore, the prerequisite for ACL all-inside reconstruction is to have a dedicated tibial guide ring and a flippable wing retrograde drill. To overcome the unfamiliarity of surgeons to apply the specific tibial guide ring in the all-inside ACL reconstruction technique by Lubowitz et al.,^17^ we described a self-made concentric cannula to combine the traditional ACL guide ring for full-length tibial tunnel construction^18^ and the guide pin sleeve in the specific guide ring for tibial socket construction.^17^ In this way, the selection of a guide ring for tibial tunnel positioning in the all-inside ACL reconstruction technique can be adjustable according to the usage habits and proficiency of the surgeons, making it easier for beginners to master the all-inside ACL reconstruction technique (Table 2). Besides, the guiding of tunnel drilling with a 2-mm Kirschner wire and the traditional tibial guide ring is better to preserve the bony integrity, if the tunnel has to be adjusted, compared with initially constructing the tunnel using a 3.5-mm retrograde drill with the specific tibial guide ring (Table 2). One drawback of our technique is that an extra self-made concentric cannula is inevitable to ensure the accurate direction of retrograde drilling (Table 2). Overall, the construction of the tibial socket in the all-inside ACL technique requires careful attention to detail and precise surgical technique. Our technique uses a self-made concentric cannula to combine the Kirschner wire with the guide pin sleeve, thereby impacting the guide pin sleeve into the tibial cortex at a controlled depth, and then retrograde drilling is performed to create the socket. This technique provides feasible approaches for surgeons to transit from traditional full-length tunnel creation to the semi-long socket construction for all-inside ACL technique.

**Table 2:** Advantages and Disadvantages of the Simplified All-Inside Tibial Socket Construction

Advantages	Disadvantages
1. The application of the Kirschner wire for the portal guide allows for the adjustment of the tibial portal position several times.	An additional 2- to 3.5-mm concentric cannula needs to be prepared.
2. The 2-mm Kirschner wire for the initial positioning avoids the cortical defect that may occur after the inaccurate positioning of the guide pin sleeve-guided 3.5-mm retrograde drilling.	
3. The replacement of the specific tibial guider for the placement of the guide pin sleeve with a traditional ACL tibial positioner has lower requirements for surgical instruments and is easier for beginners to master the all-inside ACL reconstruction technique.	

ACL, anterior cruciate ligament.

## Disclosures

All authors (Y.P., W. Yang, W. Yu, C.M., H.W., W.H) declare that they have no known competing financial interests or personal relationships that could have appeared to influence the work reported in this paper.

## Supplementary Data

Video 1 Surgical technique video of a simplified technique for tibial socket construction. The anterolateral and anteromedial portal is used as viewing portal with the patient supine and the right knee in 90° flexion for the construction of the tibial socket and 120° flexion for the femoral socket.
